# Supplementary material for: Resolving the full spectrum of human genome variation using Linked-Reads
Source: Genome Res. 2019 Apr;29(4):635–45. doi: 10.1101/gr.234443.118 (PMC6442396; doi:10.1101/gr.234443.118)
Supplement: Supplemental Material [file supp_gr.234443.118_Supplemental_longranger-2.2.zip › longranger-2.2.tar.bz2!/lr2.2-public/lib/stan/cmdstan/stan/doc/api/html/prob_2constants_8hpp_source.html]

Stan: src/stan/prob/constants.hpp Source File


|  |  |
| --- | --- |
|  | Stan  2.5.0  probability, sampling & optimization |


- Main Page
- Related Pages
- Namespaces
- Classes
- Files

- File List
- File Members

All Classes Namespaces Files Functions Variables Typedefs Enumerations Enumerator Friends Macros Pages

- src
- stan
- prob

constants.hpp

Go to the documentation of this file.

1 #ifndef STAN\_\_PROB\_\_DISTRIBUTIONS\_CONSTANTS\_HPP

2 #define STAN\_\_PROB\_\_DISTRIBUTIONS\_CONSTANTS\_HPP

3

4 #include <boost/math/constants/constants.hpp>

5

6 namespace stan {

7

8  namespace prob {

9

10

11  namespace {

12

13  const double LOG\_PI = std::log(boost::math::constants::pi<double>());

14

15  const double LOG\_ZERO = std::log(0.0);

16

17  const double LOG\_TWO = std::log(2.0);

18

19  const double NEG\_LOG\_TWO = - LOG\_TWO;

20

21  const double NEG\_LOG\_SQRT\_TWO\_PI

22  = - std::log(std::sqrt(2.0 \* boost::math::constants::pi<double>()));

23

24  const double NEG\_LOG\_PI = - LOG\_PI;

25

26  const double NEG\_LOG\_SQRT\_PI

27  = -std::log(std::sqrt(boost::math::constants::pi<double>()));

28

29  const double NEG\_LOG\_TWO\_OVER\_TWO = - LOG\_TWO / 2.0;

30

31  const double SQRT\_2 = std::sqrt(2.0);

32

33  const double LOG\_TWO\_PI = LOG\_TWO + LOG\_PI;

34

35  const double NEG\_LOG\_TWO\_PI = - LOG\_TWO\_PI;

36  }

37

38  }

39

40 }

41

42 #endif

stan::math::SQRT\_2

const double SQRT\_2

The value of the square root of 2, .

**Definition:** constants.hpp:20

stan::agrad::sqrt

fvar< T > sqrt(const fvar< T > &x)

**Definition:** sqrt.hpp:15

stan::agrad::log

fvar< T > log(const fvar< T > &x)

**Definition:** log.hpp:15


---

[ Stan Home Page ]

*© 2011–2014,
Stan Development Team.*
